# Linking micro‐X‐ray fluorescence spectroscopy and X‐ray computed tomography with model simulation explains differences in nutrient gradients around roots of different types and ages

**DOI:** 10.1111/nph.70102

**Published:** 2025-04-01

**Authors:** Eva Lippold, Magdalena Landl, Eric Braatz, Steffen Schlüter, Rüdiger Kilian, Robert Mikutta, Andrea Schnepf, Doris Vetterlein

**Affiliations:** ^1^ Department of Soil System Science Helmholtz Centre for Environmental Research – UFZ Theodor‐Lieser‐Strasse. 4 Halle/Saale 06120 Germany; ^2^ Forschungszentrum Juelich GmbH Agrosphere (IBG‐3) Juelich 52428 Germany; ^3^ Mineralogy and Geochemistry Martin Luther University Halle‐Wittenberg Von‐Seckendorff‐Platz 3 Halle/Saale 06120 Germany; ^4^ Soil Science and Soil Protection Martin Luther University Halle‐Wittenberg Von‐Seckendorff‐Platz 3 Halle/Saale 06120 Germany

**Keywords:** correlative imaging, nutrient gradients, rhizosphere, root modelling, X‐ray CT, *Zea mays*, μ‐XRF

## Abstract

- Plant roots create chemical gradients within the rhizosphere, but little information exists on the effect of root properties on the distribution of chemical gradients. The research aim was to analyse and model the effects of root type and age, radial root geometry and root hairs on nutrient gradients in the rhizosphere.
- Using micro‐X‐ray fluorescence spectroscopy (μ‐XRF) combined with X‐ray computed tomography (X‐ray CT), we analysed nutrient gradients around root segments with different diameters and ages of two *Zea mays* genotypes (wild‐type and root hair defective mutant) growing in two substrates (loam and sand). Gradients of key nutrients were compared with gradients obtained by a process‐based, radially symmetric 1D rhizosphere model.
- Results show that root hairs matter for nutrient uptake during supply limitation (phosphorus (P)), but not when it is limited by uptake kinetics (calcium (Ca), sulphur (S)). Higher Ca and S accumulation was observed at the surface of older and thicker root segments than at younger and thinner root segments.
- Micro‐XRF proved suitable for the detection of nutrient gradients of Ca and S, but not of P. While continuum modelling was well suited to explain observed nutrient gradients, it was less effective in representing pore‐related phenomena, such as precipitation reactions, which calls for new homogenization approaches.

## Introduction

The rhizosphere, defined as the area of the soil that is influenced by roots, differs fundamentally in its biochemical properties from the surrounding soil (Vetterlein *et al*., 2020). The uptake of nutrients and the transport of water to the root, as well as the release of exudates to the soil, create unique chemical gradients within the root zone (Kirk, 1999; York *et al*., 2016a; Holz *et al*., 2018b). These gradients differ in their width, shape and expression in the form of depletion or accumulation zones. Knowledge of the extent is necessary to determine the optimal root architecture in terms of exploration and exploitation, that is at which rhizosphere extent neighbouring roots influence each other (de Parseval *et al*., 2017; Landl *et al*., 2021). The extent and magnitude of gradients depend on diverse factors, such as the soil solution concentration, the nutrient uptake capacity, soil hydraulic properties, diffusion, sorption and decay (Nye, 1966; Barber, 1984; Jungk, 2001).

At present, still little is known about the relative and absolute contribution of individual factors shaping gradients around roots. A supposedly crucial parameter for the extent of physical and chemical gradients is root age (Vetterlein & Doussan, 2016), which defines the time available for interaction with the soil at a specific location (Göttlein *et al*., 1999). Some parameters influencing gradient formation also vary with root age, that is uptake rate (York *et al*., 2016b), root diameter and root hair activity (Lan *et al*., 2013). While such activity changes are well demonstrated in roots and specific root tissue in isolation (Lan *et al*., 2013), such data are much more scarce for soil‐grown plants (Kraus *et al*., 1987; Ernst *et al*., 1989). Of specific interest in terms of geometry and hence radial extent of gradients are root diameter and root hairs. Since a larger root diameter provides a larger surface area for nutrient uptake compared with a fine root, the root diameter should also have an effect on the steepness of the gradients. A fundamental role in nutrient uptake is also attributed to root hairs, which are often understood as organs shortening the distance between soil and plant surface. In this way, they improve nutrient supply, especially for strongly sorbed nutrients like phosphorus (P) (Hendriks *et al*., 1981). The effective diffusion coefficient of dissolved nutrients in soil, which is influenced by both the water content and the tortuosity of a flow path, also plays an important role, as it is the measure of the ability of a soil to conduct a certain substance through its pore space (Kuchenbuch *et al*., 1986).

The current knowledge about gradients is mostly based on data from linearized systems. Modelling shows that gradients from linear systems do not match those from radial systems. Not accounting for the radial geometry in planar experimental setups leads to an amplification of the extent and magnitude of gradients (Roose *et al*., 2016; Vetterlein *et al*., 2020). Measurements in radial systems are usually focused on cutting‐edge microscopic techniques, but with few biological replicates or restrictions imposed by roots growing in narrow tubes (Clode *et al*., 2009; Veelen *et al*., 2019).

Recently, we presented a workflow that is able to overcome these technical limitations and allows sampling in a system in which the root is growing in soil, is constricted as little as possible and still allows the measurement of typical radial geometries (Lippold *et al*., 2023). This workflow was used to observe elemental gradients in the rhizosphere of soil‐grown plants in order to understand how plants interact with the soil, and in particular, how this affects nutrient availability. The observed particle‐scale gradients were also aggregated to continuum scale 1D radially symmetric gradients around individual roots.

The objectives of the study were (1) to observe element gradients in the rhizosphere that determine plant nutrient availability of soil‐grown plants using micro‐X‐ray fluorescence spectroscopy (μ‐XRF) and (2) to test whether a 1D‐rhizosphere model is able to reproduce the experimentally observed gradients and to explain the effects of radial root geometry, root hairs and age on the gradients in two substrates reflecting a range of different soil textures. As calcium (Ca), sulphur (S) and P have been reported to potentially accumulate or deplete in the rhizosphere (Kraus *et al*., 1987; Lorenz *et al*., 1994; Hinsinger, 1998), this study was focused on these nutrients.

## Materials and Methods

### Experimental design

The pot experiment was set up as a two‐factorial, randomized design with four replicates. The term replicates here refers to individual soil columns. Factor 1 was substrate with two levels (loam and sand). Factor 2 was *Zea mays* L. genotype with two levels comprising B73 wild‐type (WT) and a root hair defective mutant (*rth3*).

### Genotypes

For the experiments, the *Zea mays* root hair defective mutant *rth3* and the corresponding WT siblings were selected (Wen & Schnable, 1994). The monogenic mutant *rth3* is transposon‐induced and shows normal root hair initiation but disturbed elongation. The mutant shows no apparent aberrant shoot phenotype, but grain yield is reduced by 20–40% compared with the WT. The mutated gene encodes a glycosylphosphatidylinositol‐anchored COBRA‐like cell wall protein RTH3 that is involved in the organization of the synthesized cellulose (Hochholdinger *et al*., 2018). The *rth3* mutants used in these experiments are genetically highly homozygous because they have been backcrossed to the inbred line B73 for more than eight generations.

### Substrates, sieving, packing and plant growth conditions

The experiment was performed as described in Lippold *et al*. (2021a,b). In brief, acrylic glass tubes of 7 cm inner diameter and 23 cm height were filled with a loamy and a sandy substrates. The substrate loam was obtained from the upper 50 cm of a haplic Phaeozem soil profile, dried to 10% gravimetric water content and sieved down to < 1 mm to create a homogeneous background in X‐ray computed tomography (X‐ray CT) images. The sand constitutes a mix of 83% quartz sand (WF 33; Quarzwerke, Weferlingen, Germany) and 17% sieved loam. Soil fertilization was done by mixing the soil with the respective salt solutions before filling. Phosphorus and Ca were added in powder form. Details on chemical and physical properties as well as the fertilization are provided in Supporting Information Tables S1 and S2. Differences in nutrient availability of both substrates were compensated for fertilization to achieve a nutrient level in the range between slightly nutrient deficient and adequate for the WT genotype (Vetterlein *et al*., 2021).

The columns were watered from top and bottom to an average volumetric water content of 22% for loam and 18% for sand, which was monitored and readjusted daily. Columns were placed in a growth chamber, which was set to 22°C during the day and 18°C at night with a 12‐h light period, 350 μM m^−2^ s^−1^ photosynthetically active radiation and a constant relative humidity of 65%. Growth duration was 21 d; that is, harvest was conducted on Day 22 after planting.

### X‐ray CT scanning

To follow root development, X‐ray tomography was performed at 7, 14 and 21 d after planting during night time to not interfere with plant photosynthesis with an industrial μCT (X‐TEK XTH 225; Nikon Metrology Europe NV, Leuven, Belgium) operated at 160 kV and 296 μA. A total of 2748 projections with an exposure time of 500 ms each were acquired during a full rotation of the columns. A lead shield was also placed between the X‐ray source and the soil column to shield the plant shoot and the soil outside of the field of view. With this setup, the dose per scan in the centre of the column amounts to 1.2 Gy (Lippold *et al*., 2021a). The obtained images were reconstructed into a 3D tomogram having a voxel size of 45 μm and an 8‐bit greyscale via a filtered back projection algorithm with the ct pro 3D software (Nikon metrology). The obtained whole‐column images were used for targeted sampling of specific root types and root ages.

### Sampling of subsamples

As described in Lippold *et al*. (2023), aluminium rings with a wall thickness of 0.25 mm and a 16 mm inner diameter and height were used for sampling. Sampling was done with a custom‐made sampling device (UGT GmbH, Muncheberg, Germany). Root segments previously identified by whole‐column X‐ray CT scans were sampled and chemically fixated using Karnovsky fixative (Karnovsky, 1965). Fixated samples were stored at 4°C until X‐ray CT analysis at 10 μm resolution, as described in Phalempin *et al*. (2021). Subsequently, all samples were dehydrated in graded acetone and embedded in Araldite 502, as described in detail in Lippold *et al*. (2023). All samples were rescanned to check for any deformations and particle displacement that had occurred during embedding. Sections with minimal structural changes were selected for further treatment. Soil sections were thinned and subsequently polished manually with a manual grinding and polishing machine (EcoMet30; Buehler, Reichshof, Germany) using diamond sanding plates of increasing fineness (MD‐Piano 80, 500, 1200, 2000 and 4000; Struers, Willich, Germany).

### Micro‐X‐ray fluorescence spectroscopy

Elemental mapping of Ca, P, S and silicon (Si) was carried out with μ‐XRF (Micro‐XRF Spectrometer M4 TORNADO; Bruker, Billerica, MA, USA). The size of the 2D region of interest was chosen, such that the root was in the centre and surrounded by 2.5–4 mm of soil to cover the anticipated gradients based on the literature (Holz *et al*., 2018a; Kravchenko *et al*., 2019; Bilyera *et al*., 2022). Whenever exact root interfaces could not be identified clearly with X‐ray CT or light microscopy, a map with a short scan time of the whole sample was done, and the combined image of Si and Ca, as well as S, was used to identify soil particles and roots, respectively. The settings for μ‐XRF were chosen as follows: Ag anode at 50 kV with 599 μA and a 20 μm spot size, stage speed of 667 μm s^−1^, equivalent to an acquisition time of 30 ms pixel^−1^. To reduce sample damage by excessive X‐ray exposure, an area of interest was mapped 10 times, and these 10 frames were accumulated to improve count statistics. Depending on the size of the region of interest and the minimum stage speed, one scan took 4–6 h.

Root diameters used for modelling were measured manually in composite S–P–Si images with imagej. Shrinkage during sample preparation could not be ruled out. To deal with shrinkage, the CT images were checked before and after embedding, and the root mask was done manually by carefully drawing the borders of the root according to the unembedded sample. Root diameters of the entire root system, as well as root length, were determined in an experiment of the same design, in which, however, the root systems were harvested as a whole in order to be able to perform subsequent analyses with WinRhizo 20 019 (Regent Instruments, Qubec, QC, Canada) on defined root orders. As an approximation for the root distribution within the experimental setup, the half‐mean distance between neighbouring roots was calculated according to Gardner (1960) and Andrews & Newman (1969).

### Soil solution

Soil solution was sampled in unplanted treatments of our experimental setup in order to inform the model about initial element concentrations at the beginning of the pot experiment. Four micro suction cups (‘MicroRhizons’; Rhizosphere Research Products B.V., Wageningen, the Netherlands) were installed in the soil at two depths (two each at 4 and 12 cm below the soil surface) to extract soil solution. Soil solution was taken 24 h after initial watering of the soil columns, and the two samples taken at each depth were combined to obtain enough solution for the element analysis. Solutions were diluted 1 : 10 in HNO_3_ (1%, Suprapur, Merck, Rahway, NJ, USA) and then analysed for element concentrations by ICP‐OES (Arcos FHS12; Spectro Analytical Instruments, Kleve, Germany).

### Image analysis

Root segmentation of the whole‐column and subsample X‐ray CT scans was carried out with a modified version of the root segmentation algorithm ‘rootine v.2’ (Phalempin *et al*., 2021). By using the μ‐XRF image of the chlorine channel, pores filled with resin were segmented within the images of the thin sections as the resin contains traces of chlorine. Roots and resin‐filled pore space were separated and segmented out manually by carefully drawing the borders of the root using the fitted chlorine maps. The pore space was masked out for Ca analysis, as the sample holder contained Ca and this signal was picked up in the pore space to different extents depending on the thickness of the thin sections. No masks and corrections were applied for the other elements. Root distances in soil were retrieved with the Euclidean distance transform of binary root images in imagej. Finally, average element counts of various μ‐XRF element maps in nonpore pixels (retrieved from segmented μ‐XRF chlorine maps) were calculated as a function of root distance (retrieved from registered 3D distance maps) with imagej.

Rhizosphere extent was calculated by adding two times the respective SD to the determined weighted average bulk soil concentrations in between 1 mm up to the maximum distance from the root measured in the respective sample. The weighting was calculated on the basis of the number of evaluable pixels in the respective root distance. Due to the particle arrangement in the sand, a simple statistical evaluation was not possible. Here, the particle arrangement evoked undular concentration gradients, as also described in Phalempin *et al*. (2021) for bulk density. The criterion for the automatic rhizosphere cut‐off was met several times with increasing distance from the root; the most likely cut‐off had to be chosen manually.

### Model description

To model the dynamics of nutrient transport and uptake in the soil around a single growing root, we set up a 1D radially symmetric rhizosphere model in COMSOL multiphysics (COMSOL Multiphysics® v.5.6., Comsol AB, Stockholm, Sweden). Following Barber (1984) and Roose *et al*. (2001), we solve the advection–diffusion–reaction model for the case of a cylindrical root in 1D while assuming linear sorption:

(Eqn 1) $$\frac{\partial c}{\partial t}\left(\theta +b\right)=\frac{1}{r}\frac{\partial }{\partial r}\left(rD_{e}\frac{\partial c}{\partial r}+aJ_{w}c\right),$$

where c is the nutrient concentration in pore water (mol cm^−3^), t is the time (s), r is the distance to the root surface (cm), $D_{e}$ (cm^2^ s^−1^) is the effective diffusion coefficient given as $D_{e}=\mathrm{D\theta f}$, where D (cm^2^ s^−1^) is the liquid diffusion coefficient, θ is the water content (cm^3^ cm^−3^), f is the diffusion impedance factor (−), which was set to the typical value of 0.3 (Van Rees *et al*., 1990), b is the soil buffer power, that is the solid‐to‐solution partition coefficient (−) of a given nutrient element (Van Rees *et al*., 1990), a is the root radius (cm), and $J_{w}$ is the water flux to the root (cm s^−1^). The equilibration between dissolved and sorbed nutrients was assumed instantaneous compared with the transport through the bulk soil, and the soil buffer power b was therefore assumed constant. Assuming a nutrient uptake of Michaelis Menten type, the boundary condition at the root surface is given as:

(Eqn 2) $$D_{e}\frac{\partial c\;}{\partial r}+J_{w}c=\frac{F_{m}c}{K_{m}+c},$$

where $K_{m}$ (mol cm^−3^) and $F_{m}$ (mol cm^−2^ s^−1^) are the Michaelis Menten constant and maximum uptake rate of the root, respectively. For better understanding, the relationship between nutrient concentration at the root surface and root nutrient uptake is illustrated in Fig. S1.

In this study, we considered a single, isolated root in a large/infinite soil domain. The experimental measurements showed half‐mean distances between neighbouring roots between 1.6 and 2 mm for the loam and 3 and 4.5 mm for the sand. Considering that the spatial extents of the rhizospheres are on average smaller than these measured half‐mean distances, we believe our assumption of an isolated root is justified, even if the occurrence of individual overlaps is possible.

We chose the radial extent of our 1D soil domain with a size of 6 cm, large enough to exclude the influence of nutrient dynamics in the rhizosphere at the soil boundary. At the soil boundary, we assumed a Dirichlet boundary with $c=c_{\mathrm{pw},\mathrm{ini}}$, where $c_{\mathrm{pw},\mathrm{ini}}$ is the initial pore water concentration.

The precipitation of CaSO_4_ from a supersaturated solution with Ca and S is considered using the experimentally found second order equation by Liu & Nancollas (1970), which is added to Eqn 1 as an additional sink term:

(Eqn 3) $$\frac{\partial c}{\partial t}\left(\theta +b\right)=\frac{1}{r}\frac{\partial }{\partial r}\left(rD_{e}\frac{\partial c}{\partial r}+aJ_{w}c\right)-kM^{2},$$

where k is the reaction rate constant (cm^3^ mol^−1^ s^−1^) and M (mol cm^−3^) is the concentration of CaSO_4_ to be deposited before equilibrium is reached and can therefore be expressed as:

(Eqn 4) $$M=\max\left(\min\left(\left(c_{\mathrm{Ca}}-{K_{\mathrm{sp}}}^{0.5}\right),\left(c_{S}-{K_{\mathrm{sp}}}^{0.5}\right)\right),0\right),$$

where $c_{\mathrm{Ca}}$ and $c_{S}$ is the pore water concentration of Ca and S, respectively, and $K_{\mathrm{sp}}$ is the solubility product of CaSO_4_. Precipitation of CaSO_4_ thus occurs if both the pore water concentrations of Ca and S are greater than the maximum solubility concentration. Considering that the rate constant of precipitation reaction to dissolution reaction of CaSO_4_ is *c*. 40 (Liu & Nancollas, 1970; Nancollas *et al*., 1979; Haghtalab *et al*., 2015), we assume that the precipitation reaction of CaSO_4_ is irreversible. It must be noted that in natural soil, co‐precipitation of CaSO_4_ with other salts, such as CaCO_3_, will occur, which significantly alters precipitation kinetics (Zarga *et al*., 2013; McFadden *et al*., 2015). However, in this study, any co‐precipitation was neglected, so the precipitation of CaSO_4_ we calculated can be considered as the maximum limit of precipitation.

To simulate the potential impact of root hairs on nutrient gradients in the rhizosphere, we adopted the ‘model 1’ approach described by Leitner *et al*. (2010). Nutrient uptake by root hairs is thereby described by an additional sink term in Eqn 1 that is only valid within the root hair zone:

(Eqn 5) $$\frac{\partial c}{\partial t}\left(\theta +b\right)=\frac{1}{r}\frac{\partial }{\partial r}\left(rD_{e}\frac{\partial c}{\partial r}+aJ_{w}c\right)-Q_{\mathrm{rh}},$$

and computed as:

(Eqn 6) $$Q_{\mathrm{rh}}=\frac{L}{l}\frac{2{\mathrm{\pi a}}_{h}}{l^{2}}\left(\frac{F_{\mathrm{mh}}c}{K_{\mathrm{mh}}+c}\right),$$

where $a_{h}$ is the root hair radius (cm), l is the distance between two root hairs (cm) and was approximated by the square root of the root surface area that is associated with a single root hair as $l=\sqrt{2a_{h}\pi /N}$, where N is the mean root hair density per unit root length, L is the characteristic root length (cm), c is the pore water concentration of the respective nutrient, and $K_{\mathrm{mh}}$ and $F_{\mathrm{mh}}$ are the Michaelis Menten constant and maximum uptake rates of the root hairs, respectively. The term L/l comes from homogenization theory and allows considering the small‐scale root hair functions at the larger single root scale (Leitner *et al*., 2010). As we are not aware of any published data on the nutrient uptake parameters of individual root hairs, we use the same values for root hairs as for the main roots, that is $K_{\mathrm{mh}}=K_{m}$ and $F_{\mathrm{mh}}=F_{m}$, as in Leitner *et al*. (2010) and Zygalakis *et al*. (2011). To show the effect of differences in root hair and root uptake parameters on nutrient gradients in the rhizosphere as well as on plant nutrient uptake, we also included a simulation scenario in which the maximum uptake rate of the root hairs is half the maximum uptake rate of the roots, i.e. $F_{\mathrm{mh}}=0.5\times F_{m}$. Diffusion impedance due to the presence of root hairs was neglected because the root hair density was low with ahl≪1. We therefore assumed that the effective diffusion in the root hair zone was equal to that in the soil. The root hair sink term $Q_{\mathrm{rh}}$ was effective only in the area of the soil where the distance to the root surface was smaller than the mean root hair length *l*_*rh*_ (cm).

### Model parameterization and scenario setup

All parameters used in the model were either directly measured in the experiment or taken from the literature and are found in Tables 1 and 2. The total initial concentrations *c*_tot,ini_ of Ca, S and P, which are assumed to be the sum of the dissolved and adsorbed concentrations, were not measured directly but had to be calculated from the soil analysis data before fertilization and the fertilization data. The total initial concentrations *c*_tot,ini_ of Ca and S were calculated from the total Ca and S contents present in the two soils given in Table S2 and from the fertilization data given in Table S1. The total initial concentration of P was calculated from the amount of plant‐available P present in the two soils and from the fertilization data, also given in Tables S1 and S3. Initial pore water concentrations, that is the dissolved concentrations, *c*_pw,ini_, were then derived as:

(Eqn 7) $$c_{\mathrm{pw},\mathrm{ini}}=\frac{c_{\mathrm{tot},\mathrm{ini}}}{\theta +b}.$$

**Table 1:** Measured parameters used in the rhizosphere model for *Zea mays*.

Parameter	Description	Value		Unit
		Loam	Sand	
*a*_*r*,primaries_	Primary root radius	0.05	0.05	cm
*a*_*r*,tips_	Tip root radius	0.0095	0.0445	cm
*θ*_mean_	Mean volumetric water content	0.22	0.18	cm^3^ cm^−3^
*R*L_tot_	Total root length	10 917	3388	cm
RWU_21_	Root water uptake between Day 20 and Day 21 (48 h)	73.3	68.6	cm^3^ d^−1^
*J*_w_	Water flux to the root (daily mean)	5.38 × 10^−7^	1.04 × 10^−6^	cm s^−1^
*c*_tot,ini,Ca_	Total initial concentration, calcium	1.27 × 10^−4^	7.47 × 10^−6^	mol cm^−3^
*c*_tot,ini,S_	Total initial concentration, sulphur	1.37 × 10^−5^	5.65 × 10^−6^	mol cm^−3^
*c*_tot,ini,P_	Total initial concentration, phosphorus	2.99 × 10^−6^	4.07 × 10^−6^	mol cm^−3^
*l*_rh_	Mean root hair length	230	244	μm
*N*	Mean root hair density per unit root length	165	164	cm^−1^

**Table 2:** Parameters from the literature used in the rhizosphere model for *Zea mays*.

Parameter	Description	Value			Unit	Source
		Calcium	Sulphur	Phosphorus		
*D*	Liquid diffusion coefficient	7.92 × 10^−6^	6.00 × 10^−6^	6.90 × 10^−6^	cm^2^ s^−1^	Samson *et al*. (2003), Iversen & Joergensen (1993), Kirk (1999)
*b*_loam_	Buffer power in loam	2	2	239	–	Barber (1984), Anghinoni & Barber (1980)
*b*_sand_	Buffer power in sand	0.3	0.35	41.3	–	Computed from *b*_loam_
*F*_m_	Maximum uptake rate	1.00 × 10^−12^	3.00 × 10^−13^	3.26 × 10^−12^	mol cm^−2^ s^−1^	Roose *et al*. (2001)
*K*_m_	Half saturation concentration	4.00 × 10^−6^	1.00 × 10^−8^	5.80 × 10^−9^	mol cm^−3^	Roose *et al*. (2001)
*k*_reaction_	Reaction rate constant, CaSO_4_	2			l mol^−1^ min^−1^	Liu & Nancollas (1970)
Ksp_CaSO4_	Solubility product of CaSO_4_ at 25°C	2.40 × 10^−5^			mol^2^ l^−2^	Meijer & Rosmalen (1984)
*rh*_lifetime_	Life time of root hairs	2			d	Jungk (2001)
*a*_h_	Root hair radius	0.0004			cm	Leitner *et al*. (2010)
*L*	Characteristic root length	1			cm	Leitner *et al*. (2010)

Soil buffer power values *b* for the three different nutrients Ca, S and P were not directly measured for the given experiment. The literature values for loam soil were taken from Barber (1984) and Anghinoni & Barber (1980). The values for the sandy substrate were estimated as follows: Soil buffer power is known to correlate with soil clay content because of its relationship to surface area (Olsen & Watanabe, 1957; Stuanes, 1982). Assuming that the buffer power is directly proportional to the clay content, we calculated the buffer power in sand from the buffer power in loam and the clay contents of the two soils. To verify that the values we assumed for buffer power were in the correct range, we compared the measured concentrations in the soil solution of the unplanted treatments with the initial pore water concentrations that we calculated with Eqn 7. For S and P, the measured and calculated pore water concentrations were in the same order of magnitude, and it can therefore be assumed that the buffer power values are in the correct range. For Ca, the measured pore water concentrations were one order of magnitude higher than the calculated ones. We therefore assumed a lower buffer power for Ca in both loam and sand so that measured and calculated pore water concentrations matched (Table S3).

For simplicity, we assumed that each segment of the root system takes up the same amount of water cm^−2^ of root surface area. The water flux to the root $J_{w}$ (cm s^−1^) was computed by Eqn 8 from the root water uptake between Day 20 and Day 21 (${\mathrm{RWU}}_{21}$ (cm^3^ s^−1^)), the total root length on Day 21 (${\mathrm{RL}}_{21}$ (cm)) and the surface of a unit root segment with mean root radius $a_{\text{mean}}$ (cm).

(Eqn 8) $$J_{w,0}=\frac{{\mathrm{RWU}}_{21}}{{\mathrm{RL}}_{21}\times 2a_{\text{mean}}\times \pi },$$

Plant water uptake was thus assumed to increase with root system growth, while $J_{w}$, the water flux to the root, was assumed as constant throughout the simulation period. To account for daily variations in root water uptake, we considered daily sinusoidal variations in the water flux to the root as:

(Eqn 9) $$J_{w}\left(t\right)=J_{w,\text{daily}}\left[\sin\left(\frac{2\mathrm{\pi t}}{24}-\frac{\pi }{2}\right)+1\right],$$

where *t* (h) is the time after midnight. Due to differences in total root length, total root water uptake and root radius, the calculated water flux to the root was around twice as large in sand as in loam. The hairless mutant *rth3* showed significantly less root length and root water uptake than the WT (Table S4); the water flux to the root $J_{w}$ (Eqn 8), however, was similar for both genotypes in both substrates. To allow a better comparison of the effects of root hairs on root nutrient uptake, we therefore kept the water flux to the root the same for the simulation scenarios with the two different genotypes WT and *rth3*.

Following Meijer & Rosmalen (1984), we assumed a solubility product constant of 2.4 × 10^−5^ mol^2^ l^−2^ for the precipitation of CaSO_4_ (Table 2). Precipitation of CaSO_4_ therefore only occurs if both the concentration of Ca and S are above the maximum solubility concentration of 4.9 mM soil solution.

It must be noted that the nutrient gradients determined by μ‐XRF are a representation of the history of nutrient dynamics between the soil and the grown root. Therefore, it can be assumed that the influence of root hairs on the measured nutrient gradients is limited by the lifetime of the root hairs. We thus assumed a functional lifetime of root hairs of 2 d (Jungk, 2001). In our simulations, we then assumed that the root hair sink term was only effective for simulation times less than the lifetime of the root hairs. To date, it is not clear how root hair density varies among different root types in a maize root system. In an experimental study on rice roots, Nestler *et al*. (2016) found similar root hair densities per unit root length on primary and lateral roots. Consistent with these results, we kept the root hair density per unit root length constant regardless of root type.

In agreement with the μ‐XRF experiment, we ran simulation scenarios over 21 d, the assumed age of the primary roots, and over 7 d, the maximum age of the root tips based on the CT images. The primary roots had a larger mean radius than the root tips, and this difference was much larger in loam than in sand. Since there is no secondary root growth in maize, root radii were kept constant throughout the simulation period. To better compare simulation results, we simulated additional scenarios with 21‐d‐old root tips with small radius and 7‐d‐old primaries with large radius in addition to the experimental setup (7‐d‐old small‐radius root tips and 21‐d‐old large‐radius primaries). In total, we ran 12 different simulation scenarios: for the two soils, loam and sand, for the two genotypes, WT (with root hairs) and *rth3* (hairless mutant), and for two root segments with different age and radius. For the WT genotype, we ran simulation scenarios with two different maximum root hair uptake rates $F_{\mathrm{mh}}$.

Nutrients are present in the soil in different states: dissolved, adsorbed and precipitated in the case of Ca and S, and dissolved and adsorbed in the case of P. Sorbed concentrations were computed from pore water concentrations as $c_{s}=c_{\mathrm{pw}}\times b$. In the following sections, all concentrations are always given per soil volume to ensure comparability. Micro‐XRF imaging qualitatively visualizes the amount of adsorbed nutrients and precipitated nutrient products, but not dissolved nutrients, as samples are dehydrated before analysis. We therefore assume that experimentally observed nutrient gradients in the rhizosphere should be compared with the simulated sum of adsorbed and precipitated concentrations for Ca and S and adsorbed concentrations for P.

It is difficult to define the extent of the rhizosphere because the transition between rhizosphere and bulk soil is gradual. Following Bilyera *et al*. (2022), the artificial boundary between the rhizosphere and bulk soil was defined by a threshold concentration for each of the nutrients considered. The rhizosphere was then defined as the area around the root where the nutrient concentration deviated from the bulk soil nutrient concentration by more than the threshold, which we set to the arbitrary value of 0.8 μmol cm^−3^ for all nutrients. This rule necessarily deviates from the rule set up for the elemental maps above, as there is no meaningful SD around the mean bulk soil concentration for a 1D rhizosphere model with a homogeneous soil domain. However, with our arbitrarily chosen threshold, the simulated rhizospheres were greater than zero but had a similar low extent as the experimentally measured ones.

### Statistics

Mean values and SE refer to four replicates. A log transformation was used before statistical analyses if normal Q–Q plots and the Shapiro test indicated that the normal distribution criterion was not met. The software originpro 2018g was used, as well as the software R v.4.2.2 (R Core Team, 2022) together with the libraries readxl (Wickham & Miller, 2023), stringr (Wickham, 2023), ggplot2 (Wickham, 2016) and ggstance (Henry *et al*., 2020). A two‐factorial ANOVA for the fixed factors substrate, genotype and their interactions was conducted in conjunction with Tukey's HSD test.

## Results

### Experimental data

**Fig. 1:**
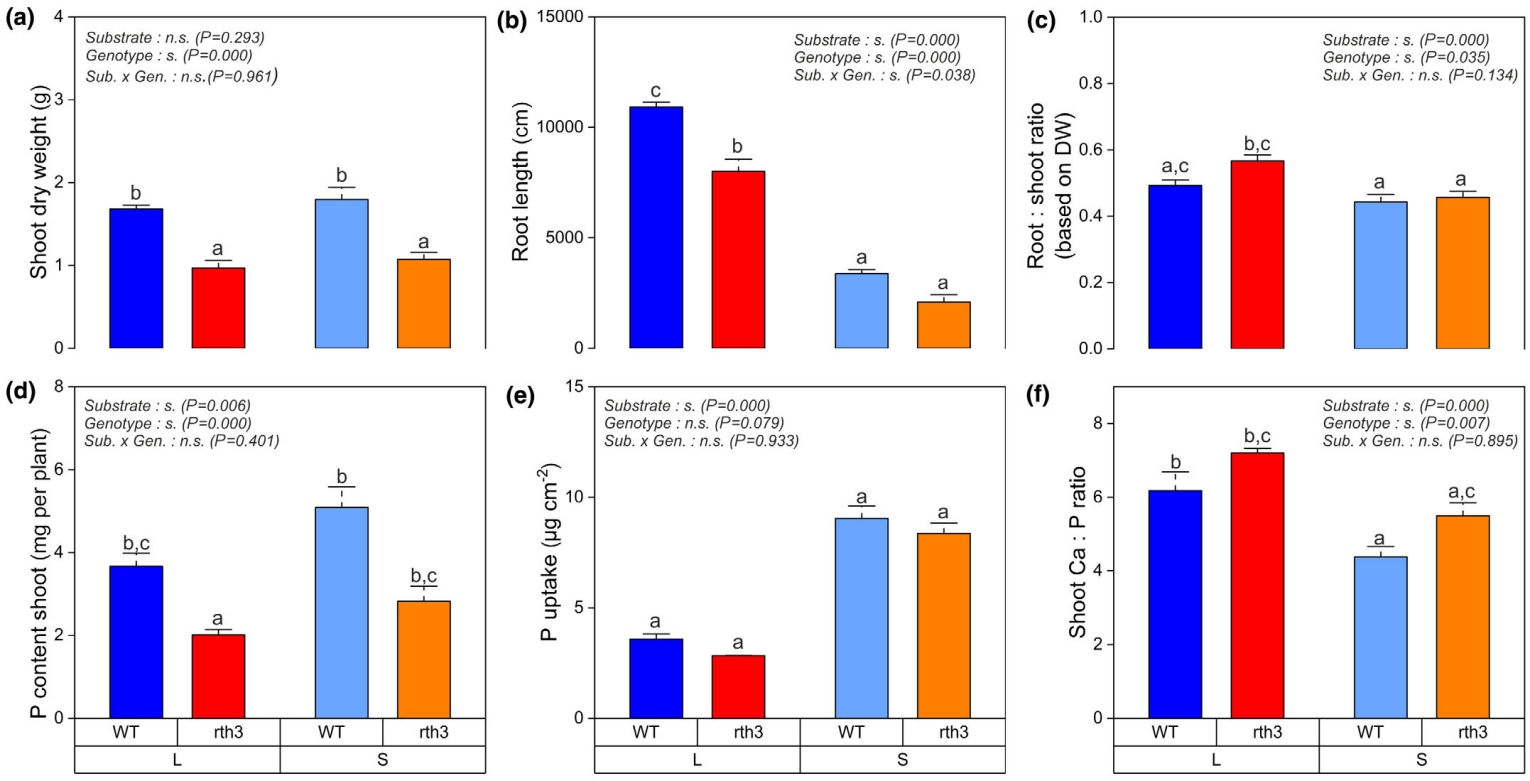
Impact of substrate (L, loam; S, sand) and *Zea mays* genotype (WT, wild‐type; *rth3,* root hair defective mutant) on shoot dry weight (a), root length (b), root : shoot ratio (c), shoot phosphorous (P) content (d), P uptake per unit root surface (e) and the stoichiometric ratio of the mobile element calcium (Ca) over the immobile nutrient P (f) in the shoot 22 d after planting. A significant effect of a factor is indicated by ‘s.’ for substrate, genotype and × for interaction (*P* < 0.05). Differences between treatments (*P* < 0.05) are indicated by different lowercase letters. Whiskers indicate SE (*n* = 4). A two‐factorial ANOVA for the fixed factors substrate, genotype and their interaction was conducted in conjunction with Tukey's HSD test. ns, non‐significant effect.

The results in terms of general growth parameters and nutrient status of the plants were similar to those from a previously published experiment (Lippold *et al*., 2021b). In general, the plants in this experiment grew slightly larger and developed a slightly higher root length in loam. We observe no significant influence of the substrate on the shoot dry weight (Fig. 1a) but on root length (Fig. 1b). A higher investment in root growth to compensate for the lack of absorbing surface provided by root hairs was found (Fig. 1b). The differences in the root : shoot ratio are not as pronounced as in previous experiments, although the ratio is still higher in loam than in sand (Fig. 1c). Growth reduction (shoot and root) was larger for loam than for sand, and the differences between genotypes were even more obvious for plant P content, with higher P content in WT (Fig. 1d). The P uptake per unit root surface of both genotypes was significantly higher in sand than in loam but did not differ between the genotypes (Fig. 1e). The Ca : P ratio showed a tendency towards higher values for *rth3* as compared to WT for loam. However, no difference between genotypes was found (Fig. 1f).

### Nutrient gradients in the rhizosphere – experimental measurements with μ‐XRF

**Fig. 2:**
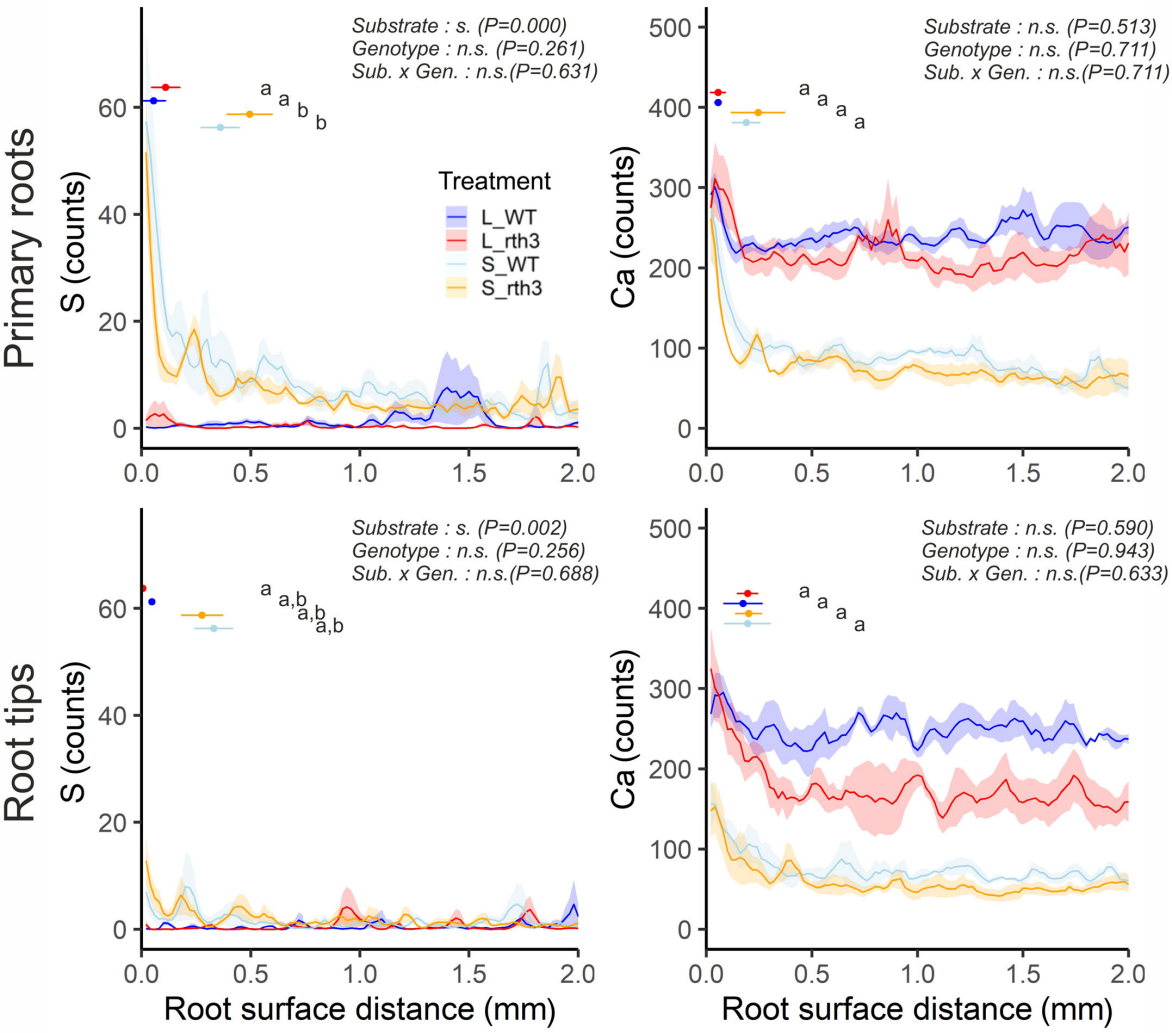
Micro‐X‐ray fluorescence spectroscopy (μ‐XRF) measurements of primary roots (upper row) and root tips of *Zea mays* showing sulphur (S) and calcium (Ca) distance‐dependent photon counts for four replicates per treatment. Shaded areas indicate SE. Points and horizontal bars show the average and SE of rhizosphere extent (*n* = 4). Two‐factorial ANOVA in conjunction with Tukey's HSD test was conducted for each rhizosphere extent. A significant effect of a factor is indicated by ‘s.’ for substrate, genotype and × for interaction (*P* > 0.05). The corresponding *P*‐values are given beside. Differences between treatments (*P* < 0.05) are indicated by different lowercase letters. ns, non‐significant effect.

**Fig. 3:**
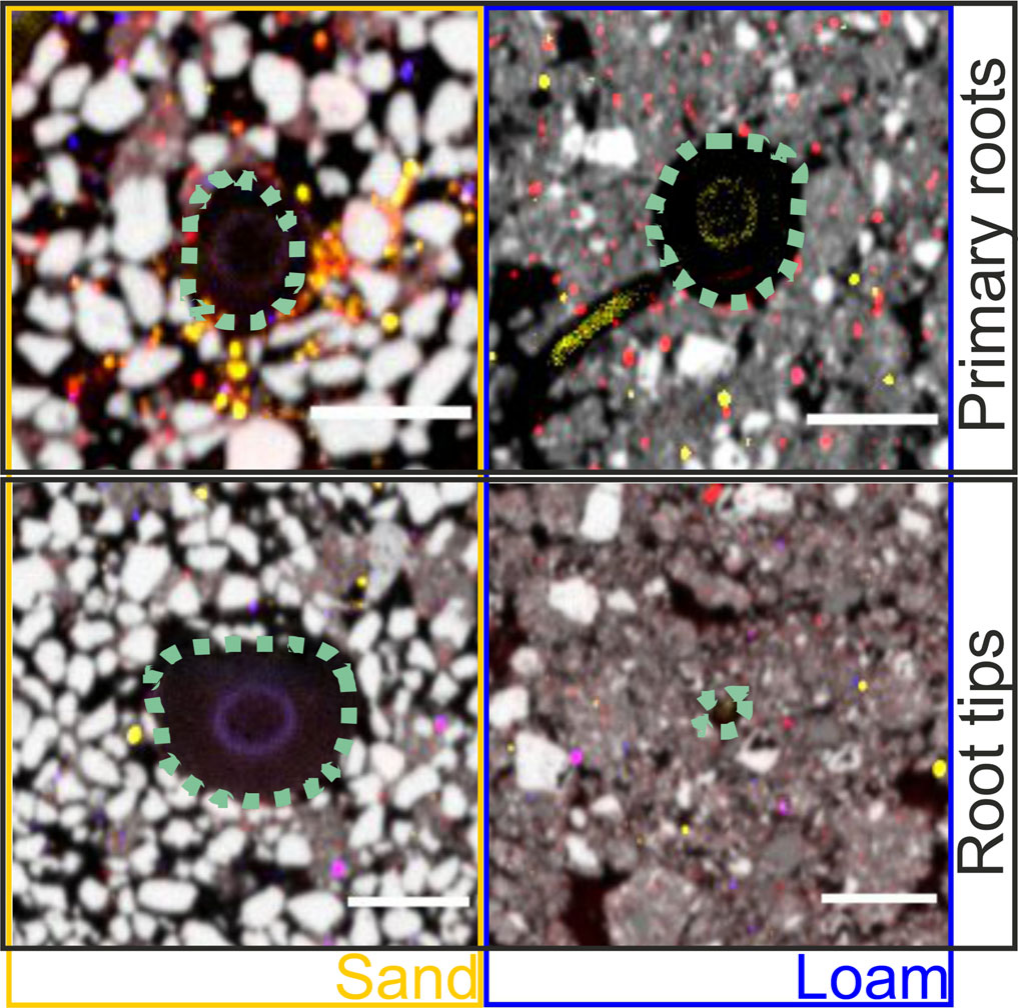
Exemplary images of primary roots and root tips of *Zea mays* in sand and loam with enrichments of sulphur (S, yellow), calcium (Ca, red) and phosphorus (blue), as well as particles containing silicon (grey). Root surface is shown in dashed line. The bar in the insets depicts 1 mm. A superposition of Ca and S in many of these spots in orange suggests a precipitation of CaSO_4_.

Experimental data revealed an accumulation of Ca and S near the root surface (Fig. 2). By contrast, no P accumulation or depletion was evident in the rhizosphere due to very poor count statistics of the μ‐XRF signal in soil, which were occasionally superimposed by high P counts of fertilizer granules or P‐bearing minerals (Fig. S2). The Ca and S gradients are largely congruent in low‐sorbing sand, whereas S accumulation in highly sorptive loam was absent despite Ca accumulation. Irrespective of the substrate, elemental maps demonstrate that the element accumulation is patchy and concentrated in spots. A superposition of Ca and S in many of these spots suggests a precipitation of CaSO_4_ (Fig. 3).

In sand, the magnitude of Ca and S accumulation in terms of concentration was greater around older roots than around root tips, whereas Ca accumulation in loam did not change with root age. Determining the spatial extent of Ca and S accumulation was not trivial due to the patchy nature at the pore scale. In sand, the extent amounted to 150–300 μm for Ca and 200–400 μm for S irrespective of genotype and root age. The distance‐dependent concentrations of individual maps exhibited an undulating pattern imprinted by the position of sand grains that occasionally vanished when averaged across four replicates with slightly different particle arrangements. This periodic enrichment pattern is completely absent in the loamy samples due to the more fine‐textured substrate and the higher background levels of Ca. The rhizosphere extent in terms of Ca accumulation was similar in loam and sand and amounted to 150–300 μm for young roots. Visual inspection suggested a similar extent for old roots, but the quantitative SD‐dependent method described in the image analysis section resulted in shorter extents due to greater variation in the bulk soil from which the SD is derived. Note that for Ca, a gradient in the range of the one for S is visible by eye but not detectable with statistics due to the high variability in bulk soil. The same occurs for Ca around primary roots in loam, where high bulk soil variability leads to a very small rhizosphere extent. Due to the patchy accumulation in a heterogeneous matrix, it is difficult to robustly describe rhizosphere extent, even if the methodology itself would allow for it. For all combinations of substrate, root type and element, the presence or absence of root hairs had no significant effect on rhizosphere extent.

The images also show a roughly similar diameter of the primary root in both substrates, but different diameters of the root tips of lateral roots. Here, the diameter of the tips in the sand sometimes even exceeds that of the primary root. This effect was confirmed in an additional experiment. While the embryonic roots are almost not influenced by the substrate, the later‐formed roots, together with their lateral roots, show a thicker diameter in sand. This cannot be explained only by a changed proportion of lateral roots or roots of different hierarchy (Fig. S3).

### Nutrient gradients in the rhizosphere – model simulation

An overview of the magnitudes of dissolved, adsorbed and precipitated nutrients present in the soil at a distance of 1 cm from the root surface in the different simulation scenarios is shown in Fig. S6. Our simulations showed that the amount of adsorbed nutrients was greater than the amount of dissolved nutrients for all three nutrients (Ca, S and P) and in all simulation scenarios. The share of the amount of dissolved nutrients to total nutrients was thereby greater in sand than in loam due to the lower buffer capacity of sand. Precipitation of CaSO_4_ in the rhizosphere occurred in both substrates; however, the share of precipitated CaSO_4_ was much less important than the share of adsorbed Ca and S. The amount of precipitated CaSO_4_ was greater around the thicker primary roots than around the thinner root tips. This is because Ca and S accumulated more at the root surface of thicker roots than at the root surface of thinner roots (Fig. S5).

**Fig. 4:**
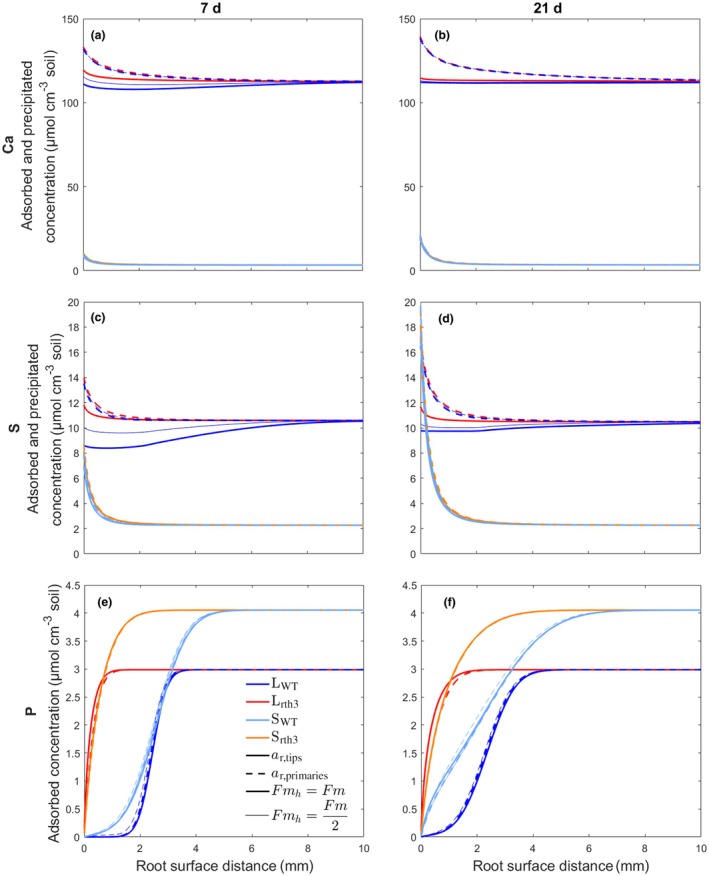
Concentration gradients of adsorbed and precipitated calcium (Ca) (a, b) and sulphur (s) (c, d) and adsorbed phosphorus (P) (e, f) around roots of *Zea mays* with different radii (*a*_*r*,tips_ and *a*_*r*,primaries_) in loam and sand for the two genotypes wild‐type (WT) (with root hairs) and *rth3* (without root hairs) after 7 and 21 d of simulation, respectively. For the WT genotype, two simulation scenarios are shown with $F_{\mathrm{mh}}=F_{m}$ and $F_{\mathrm{mh}}=\frac{F_{m}}{2}$. Note that the *y*‐axes are scaled differently in the different subplots.

Our simulations showed an accumulation of adsorbed and precipitated concentrations of Ca and S at the root surface in all different simulation scenarios except for the WT genotype in loam around the thin root tips, where root hair uptake was so high that no accumulation occurred at the root surface (Fig. 4a–d). This applied both when the maximum uptake by root hairs was set equal to the maximum uptake by the roots ($F_{\mathrm{mh}}=F_{m}$) and when the maximum uptake by root hairs was set half as high as the maximum uptake by the roots ($F_{\mathrm{mh}}=\frac{F_{m}}{2}$). For all other scenarios, this implies that the supply of Ca and S by the soil was greater than the uptake by the roots and that root nutrient uptake was thus limited by the uptake kinetics and not by the soil supply (Fig. S1).

In all scenarios, Ca and S accumulation at the root surface was higher in the soil surrounding older and thicker roots (21 d, *a*_*r*,primaries_) than in the soil surrounding younger and thinner roots (7 d, *a*_*r*,tips_). This is because Ca and S accumulated more at the root surface of thicker roots than at the root surface of thinner roots (Fig. S5) and more accumulation occurred the longer the simulation lasted. The influence of root hairs was visible in the concentration gradients of sorbed and precipitated Ca and S only around root tips in loam. In this scenario, the small root radius resulted in a high root hair density per root surface area, and the uptake by root hairs was very high. Therefore, the absence of the influence of root hairs on concentration gradients in all other scenarios is the result of a lower root hair density per root surface or, in other words, the fact that the amount of Ca and S taken up by root hairs is small compared with the amount of Ca and S transported from the soil to the root and accumulated at the root surface. Another reason is that after simulation periods of 7 and 21 d, the influence of the root hairs present for only 2 d is no longer visible. The spatial extents of the simulated Ca and S rhizospheres ranged from 700 to 9800 μm and 110 to 5200 μm, respectively, within the different scenarios. It must be noted that the spatial extents of the rhizospheres depend on the chosen threshold value, which defines how much the nutrient concentration in the rhizosphere deviates from the bulk soil nutrient concentration.

Our simulations showed a depletion of adsorbed P at the root surface in all different simulation scenarios (Fig. 4e,f). This means that P supply by the soil and not the root uptake kinetics was limiting root P uptake. Phosphorus depletion was greater in the soil surrounding older roots (21 d) than in the soil surrounding younger roots (7 d) since the longer simulation times led to greater root nutrient uptake and thus to a greater depletion of P at the root surface. Root diameter did not have a large impact on the phosphorus depletion cylinder since P uptake was supply‐limited and not uptake‐limited, and all P transported to the root was taken up immediately. Simulation scenarios with root hairs resulted in much greater P depletion in the rhizosphere due to the additional P uptake by the root hairs. The spatial extents of the simulated P rhizospheres ranged from 40 to 2090 μm within the different scenarios.

Fig. S6 gives an overview of the experimentally observed and the simulated cumulative nutrient uptake. The experiment showed that cumulative Ca and S uptake was not significantly different between WT and *rth3*, while cumulative P uptake was higher for the WT than for the *rth3*. Additionally, the experiment showed that cumulative Ca, S and P uptake was higher in sand than in loam.

In agreement with the experimental results, the model also showed higher cumulative P uptake for the WT than for the *rth3*, which suggests that root hairs are important when nutrient uptake is limited by supply. Like in the experiment, simulated P uptake was higher in sand than in loam, which can be explained by the lower buffer power of sand. Contrary to the experiment, the simulated cumulative Ca and S uptake was much higher for the WT than for the *rth3*, since we assumed that root hairs were an additional source of nutrient uptake. The difference between cumulative P uptake in WT and *rth3* was much more pronounced in the model than in the experiment. In the model, we assumed that the maximum uptake rate of root hairs is the same as or half of that of roots, respectively (Table 2). In reality, however, root hair uptake appears to be much lower than root uptake.

## Discussion

### Cumulative nutrient uptake

The comparison between experimental and model results indicated that root hairs are important when nutrient uptake is limited by supply, as was the case for P uptake, but not when nutrient uptake is limited by the uptake kinetics, as was the case for Ca and S. It can be speculated that the nutrient demand of the plant was already met by root uptake only and that the presence of root hairs, therefore, did not lead to an additional uptake.

In general, cumulative nutrient uptake was of the same order of magnitude in the experiment and model, warranting further comparisons of nutrient gradients in the rhizosphere. Overall, observed nutrient gradients in the rhizosphere may only reflect the nutrient situation around the root at a given time but provide limited information about nutrient uptake by the root.

### Nutrient gradients in the rhizosphere

The experimental results showed Ca enrichment at the root surface in both substrates and for both genotypes, as well as S enrichment at the root surface for both genotypes in sand. For P, the experiment did not show any gradients in the rhizosphere.

The simulations showed Ca and S enrichment as well as P depletion at the root surface in both substrates and for both genotypes. The observed and simulated accumulation of Ca and S in the rhizosphere can be explained by the fact that nutrient uptake was lower than soil supply, which has also been observed in other experimental studies (Lorenz *et al*., 1994; Turpault *et al*., 2007; Oliveira *et al*., 2010). One reason why no S accumulation in loam was detected in the experimental results could be imaging artefacts or a lack of sensitivity of the μ‐XRF analysis, related to leaching during embedding and differences in precipitation between substrates. Alternatively, there might be an actual process that removes sulphur from the rhizosphere in loam, which is not captured by the model. Since Ca and S are both readily soluble, we assume that they should have been affected by leaching or relocation during sample preparation (Lippold *et al*., 2023). The count intensity, which is lower for S as an element with lower photon energy than Ca, could also lead to an underdetection of S in the loam matrix. Potentially, the S signal was screened more effectively by the more abundant heavier elements in loam compared with sand. One reason for the lack of P gradients in the rhizosphere in the experimental results could be that the low counting intensity of P could have led to an underdetection of P in the heterogeneous soil matrix. Bandara *et al*. (2021) examined a sample from a previous experiment (Lippold *et al*., 2021b), but with a different embedding method. They found that the Ca distribution was almost identical to that of P. They speculated that Ca phosphates might have been deposited on the root surface. This effect was not observed in the present experiment, although P content in the plant was below the optimum according to Bergmann (1986), which could be a sign of precipitated phosphates that hinder P uptake (Jakobsen, 1979). Another explanation could be that P is present in a barely soluble form from the beginning and was therefore not available to the plant. The simulated P depletion gradients at the root surface were also found in several other experimental and model studies on P uptake by roots (Hinsinger *et al*., 2001; Jungk, 2001; Leitner *et al*., 2010; Zygalakis *et al*., 2011). This leads us to the conclusion that μ‐XRF is well suited for the detection of S and Ca, but cannot visualize nutrient gradients of lower photon intensity elements and at the same time low contents in a heterogeneous matrix, such as P.

### Spatial extents of the rhizosphere

The spatial extent of the experimentally found Ca and S rhizospheres ranged from 150 to 250 μm and 100 to 500 μm, respectively, while the spatial extent of the simulated Ca and S rhizospheres within the different scenarios ranged from 700 to 9800 μm and 110 to 5200 μm, respectively (Fig. S7). In the simulations, the large upper value of rhizosphere extents is caused by one single scenario (root tips with root hairs growing in loam), whereas all other scenarios show lower spatial extents (Fig. 4c,f). In the experiment, no S accumulation was observed in the rhizosphere of loam, irrespective of root age.

Spatial extents of measured and simulated rhizospheres cannot be compared directly with each other, as the methods for determining the extent of the rhizospheres are different. In the experiment, the extent of the rhizosphere was calculated by adding twice the SD to the determined weighted average element count in the range from 1 mm to the maximum distance from the root measured in the respective sample. In the simulation, the rhizosphere extent was defined as the area around the root where the nutrient concentration deviated from the bulk soil nutrient concentration by more than a defined threshold concentration. The magnitude of the enrichment also differs, as the calculation bases also vary. While the model refers to a given mass, the μ‐XRF measurement refers to a volume, which can vary depending on the porosity of the sample. In addition, the units of the element concentrations in the experiment and in the model simulation are different: While in the experiment, the concentration is given in element counts, and in the model results, it is given as a mass concentration. A direct conversion from counts to element concentrations is theoretically possible but challenging due to varying sample thickness and unknown matrix effects.

### Gypsum precipitation

Several studies have described CaSO_4_ precipitation in the rhizosphere due to local enrichment of Ca and S (Jaillard *et al*., 1991; Hinsinger *et al*., 2009). In the elemental μ‐XRF maps, local overlapping Ca and S accumulation spots indeed indicate CaSO_4_ precipitation. However, our model simulations showed only very little CaSO_4_ precipitation (Fig. S4). The reason for the discrepancy between experiment and model may be that our continuum model assumes a homogeneous soil and cannot account for pore‐scale phenomena, such as local nutrient enrichment, for example at grain contact points. Local nutrient enrichment spots (Fe and S) in the direct vicinity of the root surface were also shown by Veelen *et al*. (2019) using a combination of synchrotron X‐ray CT and synchrotron XRF microscopy.

### Impact of root age and diameter on nutrient gradients in the rhizosphere

In our study, measured and simulated S and partly Ca accumulation at the root surface was higher in the soil surrounding older and thicker roots than in the soil surrounding younger and thinner roots. Based on our model scenarios, this is because Ca and S accumulated more at the root surface of thicker roots than at the root surface of thinner roots, and more accumulation occurred the longer the simulation lasted. The resulting enrichment can be explained by the fact that, due to the radial geometry of the root, the soil volume from which water flows to a small‐radius root is smaller than for a large‐radius root (Fig. S5).

An additional explanation for why higher Ca accumulations were observed around young root segments in the experiment compared with the model could be that the uptake of Ca along the root has two maxima: at the root tip and a little further away where the lateral primordia had broken through the endodermis (Ferguson & Clarkson, 1975; Häussling *et al*., 1988). Ferguson & Clarkson (1975) showed that Ca is taken up by all parts of the root, but there is a decline in uptake from apex to base with a marked maximum in Ca translocation in 12 cm distance from the root tip where lateral roots are initiated in the pericycle and where the structure of the endodermis may change transiently, while at the base of the roots, the internal root development restricts Ca translocation. In the present study, samples were taken *c*. 18 cm above the root tip. Based on X‐ray CT images, we know that lateral roots were already fully developed for the primary root in this area. The described area of maximum Ca uptake on the day of harvest would be, in accordance with CT images, in 5 to 10 cm distance from the sampled region. Different uptake rates along the axis of individual roots as well as for different root types are also reported for other nutrients as well as for water (Hayward & Spurr, 1943; Ferguson & Clarkson, 1975; York *et al*., 2016b; Ahmed *et al*., 2018). In our model, however, we used constant Michaelis–Menten parameters and constant root water uptake, as we lack more precise data. Our model results showed greater P depletion in the soil surrounding older roots than in the soil surrounding younger roots, which is caused by a greater root P uptake with time, causing a greater depletion of P at the root surface and corresponds to findings from the literature (Menezes‐Blackburn *et al*., 2016).

### Impact of the different genotypes WT and *rth3* on nutrient gradients in the rhizosphere

Neither the experiment nor the model could find a significant influence of root hairs on Ca and S gradients. This is because both Ca and S uptake were limited by uptake kinetics rather than soil supply, and even if root hairs took up additional Ca and S, fast nutrient replenishment by the soil would prevent this additional uptake from being reflected in rhizosphere gradients several days after the root hairs have lost their function. From an experimental point of view, it is still not clear whether and how root hairs contribute to Ca uptake (Bienert *et al*., 2021). For S uptake, the importance of root hairs is considered proven, but there seems to be no direct relationship between root hair length and density and root S uptake (Bienert *et al*., 2021). In our model simulations, P concentration gradients in the rhizosphere were found to be strongly influenced by the presence of root hairs, consistent with findings in the literature (Jungk, 2001; Leitner *et al*., 2010). This is because root P uptake was limited rather by soil supply than by uptake kinetics. Therefore, the additional decrease in concentration gradients due to P uptake by root hairs could not be quickly compensated for by P supply through the soil.

### Impact of the bulk soil on nutrient gradients in the rhizosphere

Despite the high variability of bulk soil, also the definition of bulk soil from 1 mm up to the maximum distance measured may need to be questioned, as different rhizosphere extents for various elements are reported in the literature (Darrah, 1993; Hinsinger & Gilkes, 1996; Bilyera *et al*., 2022). These values differ from millimetre to centimetre depending on the element of interest and the way the experiments were set up. Furthermore, most of these values are based on linearized systems, which are not fully comparable to radial gradients (Vetterlein *et al*., 2020).

Another observation, which is well in line with the findings of Phalempin *et al*. (2021), and might explain the discrepancy between experiment and model, is the oscillating behaviour of counts close to the root in sand. When particles have a uniform size in well‐sorted substrates, they tend to assemble at regular distances to the root, causing successions of high and low porosity after averaging across the plane that only vanishes after a distance of several grains. In the present study, such an oscillatory behaviour for Ca and S was also observed. The reason may be that precipitation or accumulation of elements can only occur within the pore space and might preferentially occur at grain contacts. This effect cannot be described by our continuum model, which assumes a homogeneous soil domain. Evaluation of more replicates would presumably eventually smooth out the pore effect of the experimental data, in particular in loam, making it easier to see the effective expansion. If the matrix rather than the pore space is considered, density gradients around the roots should also be visible, apart from the oscillating behaviour described above.

According to Phalempin *et al*. (2021) in similar samples, roots had to create their own pores by pushing away soil particles. For samples with the same bulk density as the one presented here, a zone of lower bulk density close to the root was observed, which had an extent of a maximum 0.5 mm. Behind this zone, a zone of soil compaction ranging from 0.25 to 1 mm was observed in loam. Comparing these results with the Si images of the primary root, we observe a similar behaviour in particle arrangement for both substrates for thicker roots, which are the primary roots in our experiment. These porosity variations modulate the available space for precipitates, the number of particle contacts per volume and the gravimetric precipitate content for a given precipitate mass. In a study done with synchrotron XRF, the density gradient was included in the analysis in order to correct for the porosity effect (Veelen *et al*., 2019). The amount of elements precipitated in the pore space, relative to the mass of the soil, would be even higher in this experiment if the soil density was included. This shows a general discrepancy in the comparison between volume and mass‐related data, which emerges from the classic laboratory methods of analysing soil samples.

### Limitations of the used model approach

Modelling nutrient transport and uptake by a single root with root hairs is not trivial since the soil is not a homogeneous domain and the root scale is much larger than the root hair scale. To solve this problem, we adopted the multiscale approach by Leitner *et al*. (2010). In this approach, the homogenization method (Pavliotis & Stuart, 2008) is used to transform spatial heterogeneities at the root scale and root hair scale into a comprehensible homogeneous description. The resulting effective model of nutrient transport and uptake in the root hair zone of a single root then contains the relevant information about the root hair geometry implicitly. One advantage of this approach is that the model is generic and was not developed for a specific crop. In this work, we have focused on maize as the modelling accompanies the experimental study. To apply the model to other crops, only individual input parameters need to be adjusted.

However, soil contains additional complexity due to factors, such as the tortuosity of the pore network or the sorption of diffusing nutrients on the surfaces of the mineralogically diverse soil particles, some of which are also covered by organic matter. Our model does not account for these complex relationships and makes the simplifying assumption that the soil is homogeneous around a single root hair. For this reason, experimentally observed local precipitation of gypsum could not be adequately represented by our model simulations. To address the complexity of the soil pore space in model simulation, Zygalakis *et al*. (2011) developed a dual‐porosity model, which accounts for the impact of soil particles on diffusion. However, this model requires a large number of parameters related to the soil particle space, which were not measured in our experiment. To increase model accuracy and to additionally incorporate information regarding nutrient uptake at the soil pore level, Keyes *et al*. (2013) and Daly *et al*. (2016) used image‐based modelling. Such modelling is well suited to assess how soil surface binding responses and geometries affect nutrient uptake by root hairs, CaSO_4_ precipitation due to local accumulation of Ca and S, as well as other rhizosphere processes, and can help to further develop continuum models, such as the one used in this study. However, these models are not well suited to run simulation scenarios with different parameterizations (e.g. soil substrate, soil water content and plant genotype) because an individual X‐ray CT image is required for each scenario. In addition, a single simulation represents only the realization of a particular scenario. A different image of the same scenario could lead to different simulation results. To account for pore‐scale phenomena, such as gypsum accumulation while keeping computational costs to a minimum, a new homogenization approach is required that takes into account the complexity of the soil pore space.

### Concluding remarks

This study contributes to the existing research base by offering further insights into the extent of nutrient gradients surrounding roots. A deeper understanding of these gradients is essential, for example, to determine the optimal root architecture in terms of exploration and exploitation. Linking model and experimental data allowed us to check the plausibility of the measured data, explain observed effects and position the chemical gradients within the soil‐grown root system. Using the model, we were able to show that root hairs are important for nutrient uptake when it is limited by supply, as was the case for P, but not when it is limited by uptake kinetics, as was the case for Ca and S. We were also able to show the importance of radial root geometry, which led to greater measured and simulated S and partly Ca accumulation at the root surface of thick root segments than at the root surface of thin root segments. We demonstrated that μ‐XRF is a powerful method for detecting nutrient gradients of higher photon intensity elements, such as Ca and S, but cannot visualize nutrient gradients of lower photon intensity elements and at the same time low contents in a heterogeneous matrix, such as P. The measured precipitation of CaSO_4_ caused by large local concentrations of Ca and S is too patchy to be modelled by a continuum model that does not include pore‐scale processes. A new homogenization approach, used to transform spatial heterogeneities at the root scale into a comprehensible homogeneous description, would be required that considers the complexity of the soil pore space.

## Competing interests

None declared.

## Author contributions

EL and EB carried out the growth experiment and carried out the preparation of thin slices. EL and RK carried out the μ‐XRF measurements. DV, SS and AS acquired the funding and did the conceptualization. RM acquired the funding for μ‐XRF. EL and SS carried out the correlative imaging. ML did the modelling. EL and ML wrote the first draft of the manuscript and contributed equally to this work. All authors wrote the manuscript and have given approval for the final version of the manuscript. EL and ML are contributed equally to this work and share first authorship.

## Disclaimer

The New Phytologist Foundation remains neutral with regard to jurisdictional claims in maps and in any institutional affiliations.

## Supporting information


**Fig. S1** Relationship between concentration at the root surface and root nutrient uptake per unit length of a root segment.
**Fig. S2** μ‐XRF measurements of primary roots and root tips showing phosphorus distance‐dependent photon counts for four replicates per treatment.
**Fig. S3** Average root diameter measured in the complete root system and separately for the primary roots.
**Fig. S4** Amount of nutrients present in the soil at a distance of 1 cm from the root surface in different states.
**Fig. S5** Influence of root radius on the nutrient concentration at the root surface caused by advective transport.
**Fig. S6** Cumulative nutrient uptake per root surface area after 21 d of growth of a primary root in the experiment and in the model.
**Fig. S7** Comparison between measured and simulated nutrient concentration gradients around primary root segments.
**Table S1** Substrate specific fertilization.
**Table S2** Selected characteristics of the substrates ‘loam’, ‘sand’.
**Table S3** Measured and calculated pore water concentrations for Ca, S and P.
**Table S4** Measured total root length and root water uptake on day 21.
Please note: Wiley is not responsible for the content or functionality of any Supporting Information supplied by the authors. Any queries (other than missing material) should be directed to the *New Phytologist* Central Office.

## Data Availability

The datasets generated during and/or analysed during this study are available in the zenodo repository: doi: 10.5281/zenodo.14892860.
