# Supplementary material for: Linking micro‐X‐ray fluorescence spectroscopy and X‐ray computed tomography with model simulation explains differences in nutrient gradients around roots of different types and ages
Source: New Phytol. 2025 Apr 1;246(4):1780–95. doi: 10.1111/nph.70102 (PMC12018778; doi:10.1111/nph.70102)
Supplement: Supplementary file 1 — Fig. S1 Relationship between concentration at the root surface and root nutrient uptake per unit length of a root segment. Fig. S2 μ‐XRF measurements of primary roots and root tips showing phosphorus distance‐dependent photon counts for four replicates per treatment. Fig. S3 Average root diameter measured in the complete root system and separately for the primary roots. Fig. S4 Amount of nutrients present in the soil at a distance of 1 cm from the root surface in different states. Fig. S5 Influence of root radius on the nutrient concentration at the root surface caused by advective transport. Fig. S6 Cumulative nutrient uptake per root surface area after 21 d of growth of a primary root in the experiment and in the model. Fig. S7 Comparison between measured and simulated nutrient concentration gradients around primary root segments. Table S1 Substrate specific fertilization. Table S2 Selected characteristics of the substrates ‘loam’, ‘sand’. Table S3 Measured and calculated pore water concentrations for Ca, S and P. Table S4 Measured total root length and root water uptake on day 21. Please note: Wiley is not responsible for the content or functionality of any Supporting Information supplied by the authors. Any queries (other than missing material) should be directed to the New Phytologist Central Office. [file NPH-246-1780-s001.docx]

New Phytologist Supporting Information

Article title: Linking micro X-ray fluorescence spectroscopy and X-ray computed tomography with model simulation explains differences in nutrient gradients around roots of different types and ages

Authors: Eva Lippold^1*^, Magdalena Landl^2^*, Eric Braatz^1^, Steffen Schlüter^1^, Rüdiger Kilian^3^, Robert Mikutta^4^, Andrea Schnepf^2^, Doris Vetterlein^1,4^

^1^ Department of Soil System Science, Helmholtz Centre for Environmental Research – UFZ, Theodor-Lieser-Strasse. 4, 06120 Halle/Saale, Germany

^2^ Forschungszentrum Juelich GmbH, Agrosphere (IBG-3), 52428 Juelich, Germany

^3^ Mineralogy and Geochemistry, Martin Luther University Halle-Wittenberg, Von-Seckendorff-Platz 3, 06120 Halle (Saale), Germany

^4^ Soil Science and Soil Protection, Martin Luther University Halle-Wittenberg, Von-Seckendorff-Platz 3, 06120 Halle (Saale), Germany

*shared first authorship

*Corresponding author:*
Eva Lippold, eva.lippold@ufz.de

Article acceptance date: 23 February 2025

Figures: 7

Tables: 4


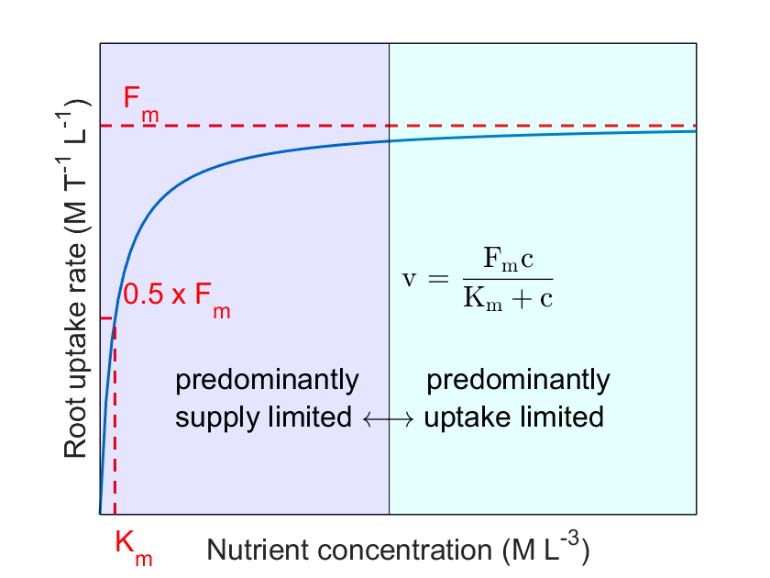


Figure S1: The relationship between concentration at the root surface and root nutrient uptake per unit length of a root segment i.e. the right hand side of equation (2) according to Michaelis-Menten, where $K_{m}$ is the Michaelis Menten constant, $F_{m}$ the maximum root uptake rate, $v$ the root uptake rate and $c$ the nutrient concentration at the root surface. If the nutrient concentration at the root surface is low and the root uptake rate increases significantly with increasing nutrient concentration (left side of the plot), root nutrient uptake is limited by the concentration at the root surface and thus by the nutrient supply by the soil. If the nutrient concentration at the root surface is high and the root uptake rate does not change much with increasing nutrient concentration (right side of the plot), root nutrient uptake is limited by the uptake kinetics, i.e. the maximum uptake rate F_m_.


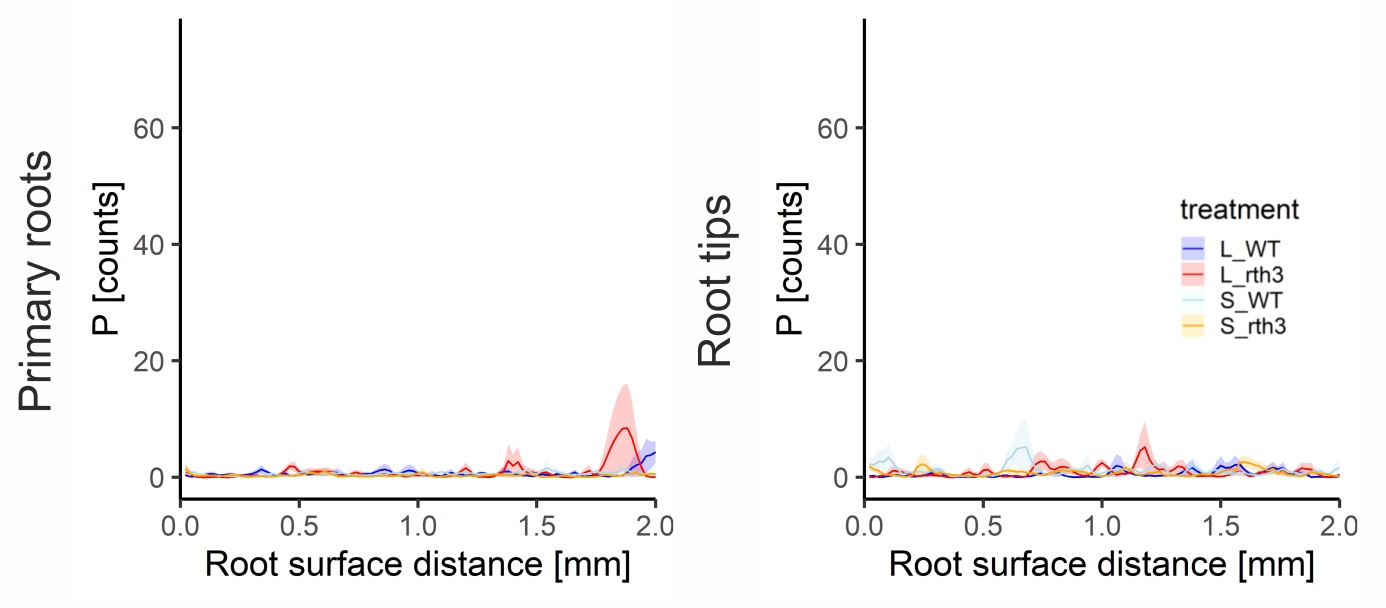


Figure S2: µ-XRF measurements of primary roots and root tips showing phosphorus (P) distance-dependent photon counts for four replicates per treatment. Shaded areas indicate standard error.


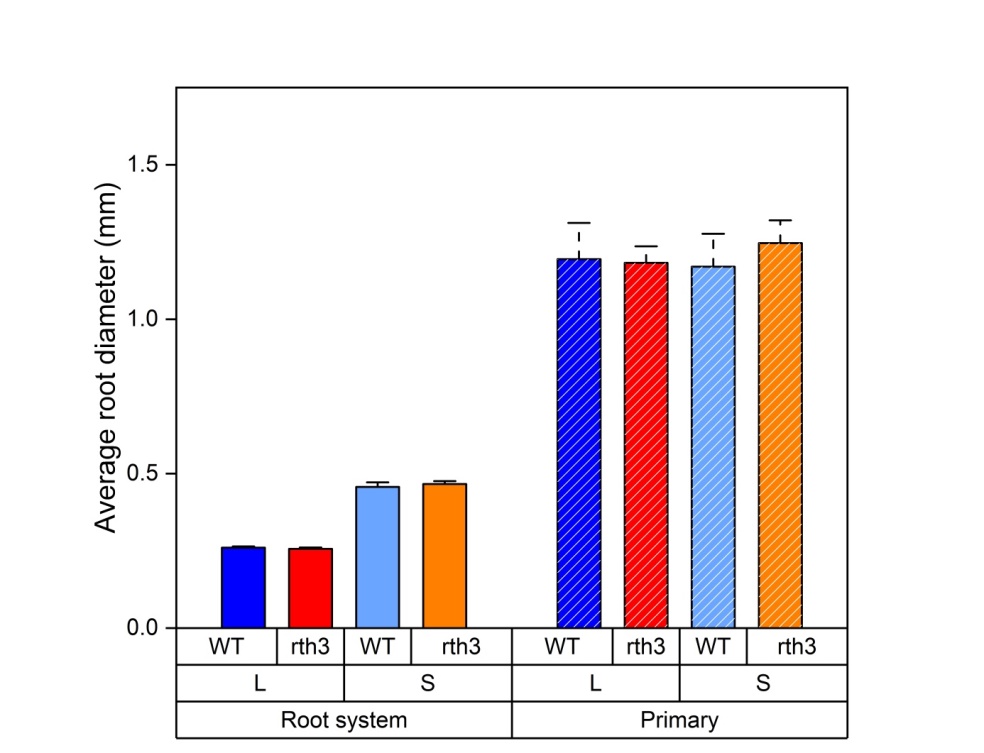


Figure S3: Average root diameter measured in the complete root system and separately for the primary roots. It can be seen that the embryonic roots are not affected by the soil texture, while the complete root system shows a reaction. Here, the diameters are higher in clay than in sand. The bars depict the mean values of four treatments with the standard error.


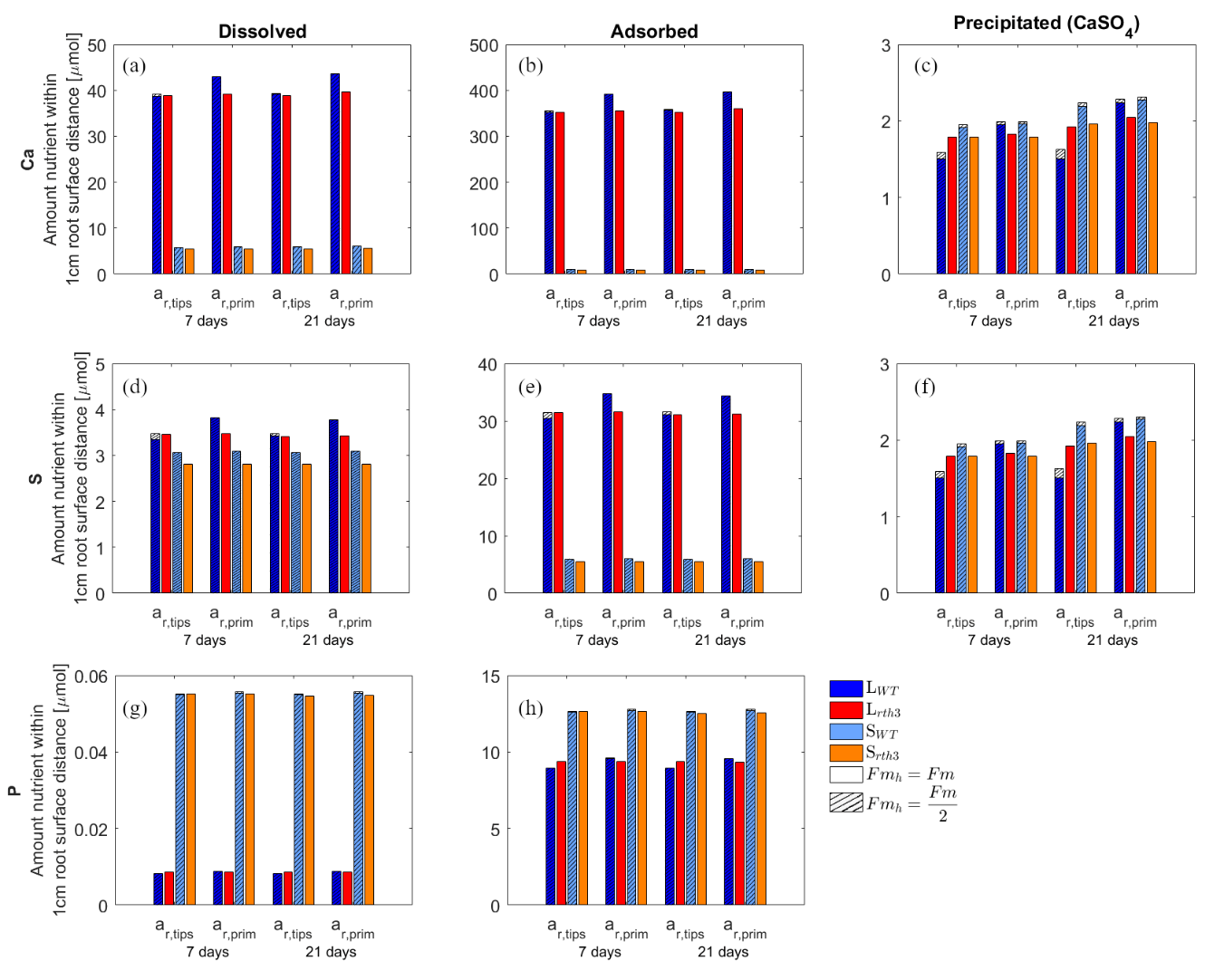
 Figure S4: Amount of nutrients present in the soil at a distance of 1 cm from the root surface in different states (dissolved (a,d,g), adsorbed (b,e,h), precipitated as CaSO_4_ (c,f)) for C (a-c), S (d-f) and P (g,h) for the two soils loam and sand, the two genotypes WT and *rth3*, the two simulation times (7d, 21d), different radii (a_r,tips_, a_r,prim_)) and two simulation scenarios with $F_{mh}=F_{m}$ and $F_{mh}=\frac{F_{m}}{2}$ for the WT genotype. It must be noted that the y-axes are scaled differently in the different subplots.


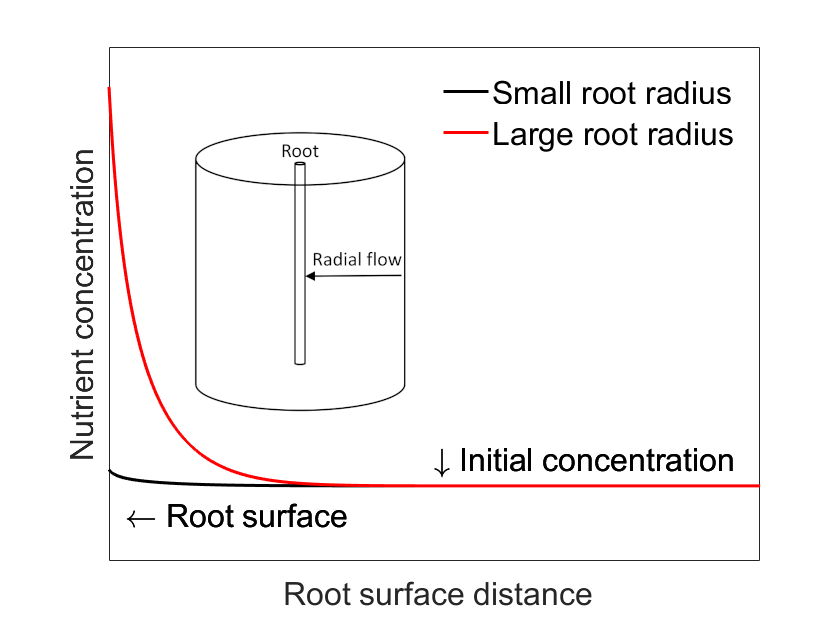


Figure S5: Influence of root radius on the nutrient concentration at the root surface caused by advective transport without consideration of root nutrient uptake in our radial single-root model. Due to the radial geometry of our model, the water flux $J_{w}$declines with radial distance from the root surface according to $J_{w(r)}={J_{w(a)}\times a}/r \left( r>a \right)$. Therefore, the soil volume from which water flows to a small-radius root is smaller than for a large-radius root, and so is the corresponding advective transport of nutrients to the root surface, which affects the nutrient concentration at the root surface. The relationship between root radius and nutrient concentration at the root surface caused by advective transport is illustrated.


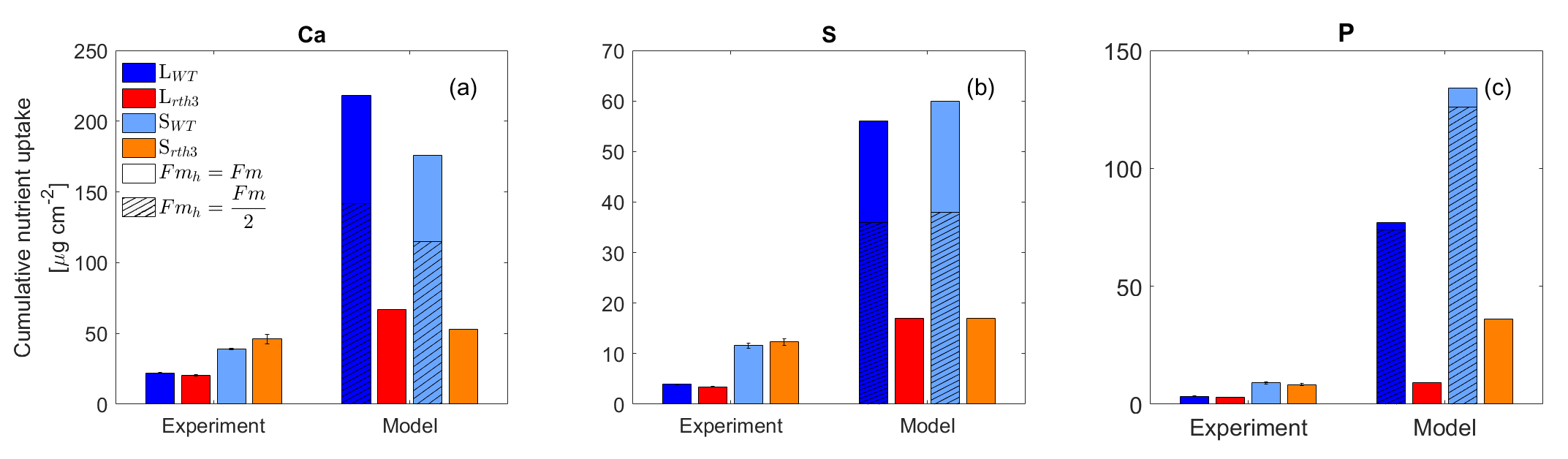


Figure S6: Cumulative nutrient uptake per root surface area [µg cm^-2^] after 21 days of growth of a primary root in the experiment and in the model in loam and sand for the two genotypes WT (with root hairs) and *rth3* (without root hairs) for Ca (a), S (b) and P(c), the experimental data show mean ± SE. In the model, two simulation scenarios with $F_{mh}=F_{m}$ and $F_{mh}=\frac{F_{m}}{2}$ are shown for the WT genotype. It should be noted that in the experiment, the cumulative nutrient uptake of the plant was normalized with the surface area of the root system on the last day of the experiment and not with the (unknown) surface area of the root system at the actual time of nutrient uptake, which means that the cumulative uptake per root surface area calculated from experimental observations is lower than in reality. In contrast, the model directly simulated the cumulative uptake per root surface area. It is therefore clear that the experimentally determined cumulative nutrient uptake per root surface area must be lower than that simulated by the model.


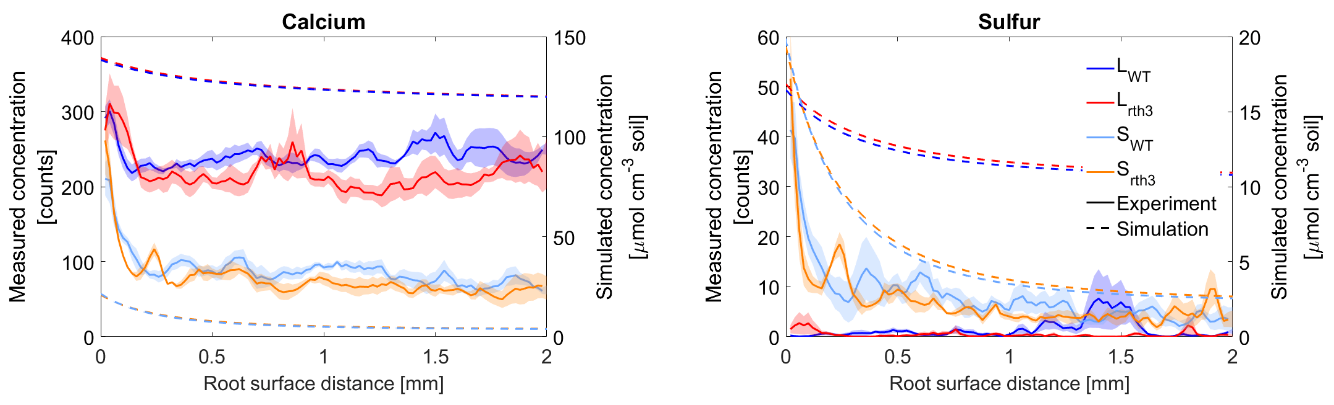


Figure S7: Comparison of measured and simulated nutrient concentration gradients around primary root segments in loam and sand for the two genotypes WT (with root hairs) and *rth*3 (without root hairs) after 21 days of growth. For the WT genotypes, only the simulation scenario with $F_{mh}=F_{m}$ is shown for better visibility.

| Table S1: Substrate specific fertilization   \| Substrate \| Nutrient \| Application rate  [mg nutrient kg^-1^] \| \| Type \| \| --- \| --- \| --- \| --- \| --- \| \| Loam \| N \| 50 \| NH_4_NO_3_ \| \| \| P \| 40 \| CaHPO_4_ \| \| \| K \| 50 \| K_2_SO_4_ \| \| \| Mg \| 25 \| MgCl_2_ x 6 H_2_O \| \| \| Sand \| N \| 100 \| NH_4_NO_3_ \| \| \| P \| 80 \| CaHPO_4_ \| \| \| K \| 100 \| K_2_SO_4_ \| \| \| Mg \| 50 \| MgCl_2_ x 6 H_2_O \| \| \| Ca \| 100 \| CaSO_4_ x 2 H_2_O \| \| \| Mn \| 3.25 \| MnSO_4_ x H_2_O \| \| \| Zn \| 0.79 \| Zn(NO3)_2_ x 4 H_2_O \| \| \| Cu \| 0.5 \| CuSO_4_ x 5 H_2_O \| \| \| B \| 0.17 \| H_3_BO_3_ \| \| \| Fe \| 3.25 \| Fe-EDTA \| \|   Table S2: Selected characteristics of the substrates ‘loam’, ‘sand’. Total element content of P, Ca, and S determined with X-ray fluorescence spectroscopy on pressed tablets.   \|  \| ‘loam’ \| ‘sand’ \| \| --- \| --- \| --- \| \| Bulk density [g cm^-^³] \| 1.26 \| 1.47 \| \| pH (CaCl_2_) \| 6.21 \| 6.25 \| \| Carbonate [g kg^-1^] \| <1 \| <1 \| \| Sand [%] \| 33.2 \| 47.7 \| \| Silt [%] \| 47.7 \| 8.1 \| \| Clay [%] \| 19.1 \| 3.3 \| \| CEC [mmol_c_ kg^-1^] \| 76.1 \| 13.0 \| \| C_org_ [%] \| 0.84 \| 0.14 \| \| N_t_ [%] \| 0.084 \| 0.014 \| \| P plant available [mg kg^-1^] \| 33.41 \| 5.67 \| \| K plant available [mg kg^-1^] \| 26.67 \| 5.44 \| \| P [mg kg^-1^] \| 960 \| 729 \| \| Ca [mg kg^-1^] \| 4000 \| - \| \| S [mg kg^-1^] \| 329 \| - \|   Table S3: Measured and calculated pore water concentrations for Ca, S, and P.   \|  \| Measured pore water concentrations [mol cm^-3^] \| \| Calculated pore water concentrations [mol cm^-3^] \| \| \| --- \| --- \| --- \| --- \| --- \| \|  \| Loam \| Sand \| Loam \| Sand \| \| Ca \| 5.55 $\times$ 10^-5^ \| 6.46 $\times$ 10^-5^ \| 5.72 $\times$ 10^-5^ \| 1.42 $\times$ 10^-5^ \| \| S \| 1.06 $\times$ 10^-5^ \| 1.43 $\times$ 10^-5^ \| 6.19 $\times$ 10^-6^ \| 1.07 $\times$ 10^-5^ \| \| P \| 1.60 $\times$ 10^-8^ \| 4.00 $\times$ 10^-8^ \| 1.25 $\times$ 10^-8^ \| 9.80 $\times$ 10^-8^ \|   Table S4: Measured total root length and root water uptake (RWU) on day 21 (mean, std) for WT and *rth3* grown in loam and sand   \|  \| Total root length  mean (std)  [cm] \| RWU, day 21  mean (std)  [cm^3^] \| \| --- \| --- \| --- \| \|  \| \|  \| \| L, WT \| 10917.4 (445.4)  7999.7 (1117.8)  3387.9 (340.3)  2082.4 (687.6) \| 73.3 (4.9) \| \| L, *rth3* \| 50.8 (7.3) \| \| S, WT \| 68.6 (14.7) \| \| S, *rth3* \| 38.0 (11.9) \| |
| --- | --- | --- | --- | --- | --- | --- | --- | --- | --- | --- | --- | --- | --- | --- | --- | --- | --- | --- | --- | --- | --- | --- | --- | --- | --- | --- | --- | --- | --- | --- | --- | --- | --- | --- | --- | --- | --- | --- | --- | --- | --- | --- | --- | --- | --- | --- | --- | --- | --- | --- | --- | --- | --- | --- | --- | --- | --- | --- | --- | --- | --- | --- | --- | --- | --- | --- | --- | --- | --- | --- | --- | --- | --- | --- | --- | --- | --- | --- | --- | --- | --- | --- | --- | --- | --- | --- | --- | --- | --- | --- | --- | --- | --- | --- | --- | --- | --- | --- | --- | --- | --- | --- | --- | --- | --- | --- | --- | --- | --- | --- | --- | --- | --- | --- | --- | --- | --- | --- | --- | --- | --- | --- | --- | --- | --- | --- | --- | --- | --- | --- | --- | --- | --- | --- | --- | --- | --- | --- | --- | --- | --- | --- | --- | --- | --- | --- | --- |
